# Hemophagocytic Lymphohistiocytosis and Progressive Disseminated Histoplasmosis

**DOI:** 10.3201/eid2206.151682

**Published:** 2016-06

**Authors:** Kenice Ferguson-Paul, Spencer Mangum, Ashley Porter, Vasiliki Leventaki, Patrick Campbell, Joshua Wolf

**Affiliations:** St. Jude Children’s Research Hospital, Memphis, Tennessee, USA (K. Ferguson-Paul, S. Mangum, A. Porter, V. Leventaki, P. Campbell, J. Wolf);; University of Tennessee Health Sciences Center, Memphis (K. Ferguson-Paul, S. Mangum, A. Porter, J. Wolf)

**Keywords:** infant, histoplasmosis, lymphohistiocytosis, hemophagocytic, rare diseases, fungi, *Suggested citation for this article*: Ferguson-Paul K, Mangum S, Porter A, Leventaki V, Campbell P, Wolf J. Hemophagocytic lymphohistiocytosis in infant with progressive disseminated histoplasmosis. Emerg Infect Dis. 2016 Jun [*date cited*]. http://dx.doi.org/10.3201/eid2206.151682

**To the Editor:** Progressive disseminated histoplasmosis (PDH) of infancy occurs most commonly in previously healthy infants <1 year of age, typically after exposure to a large fungal inoculum (*1*). Even with treatment, the disease is fatal in ≈13% of cases (*1*). Common symptoms include fever, hepatosplenomegaly, lymphadenopathy, and failure to thrive. Laboratory abnormalities frequently include cytopenia and coagulopathy (*1*,*2*). Many clinical manifestations of PDH overlap with those of the hyperinflammatory condition hemophagocytic lymphohistiocytosis (HLH), and co-existence of HLH and histoplasmosis has been reported in adults. We report a case of simultaneous PDH and HLH in an infant.

A 6-month-old African American girl was brought for treatment to St. Jude Children’s Research Hospital in April 2015 with a 1-month history of daily fever. Her history was notable only for a methicillin-resistant *Staphylococcus aureus* skin abscess diagnosed when she was 5 months old; it was drained, and she received oral clindamycin.

On initial evaluation, she had fever, lethargy, and hepatosplenomegaly. Laboratory testing showed pancytopenia and mild hepatitis, and abdominal ultrasound confirmed hepatosplenomegaly. After admission, respiratory distress developed due to worsening hepatosplenomegaly. Additional laboratory testing revealed elevated ferritin (1,218 ng/mL; reference 10–100 ng/mL), low fibrinogen level (78 mg/dL; reference 185–443 mg/dL), elevated triglycerides (378 mg/dL; reference 0–149 mg/dL) and elevated soluble interleukin-2 receptor (21,530 pg/mL; reference <1,033 pg/mL). An HIV serologic test result was negative. Histologic examination of bone marrow showed many activated macrophages, including some with hemophagocytosis (Technical Appendix). On the basis of clinical and laboratory findings, the patient received a diagnosis of HLH, and treatment was initiated with etoposide and dexamethasone.

Further evaluation of the bone marrow sample demonstrated fungal elements characteristic of *Histoplasma capsulatum* (Technical Appendix). *Histoplasma* antigen assays on serum and urine samples were positive for *H. capsulatum* above the limit of quantification. *Histoplasma* complement-fixation antibody titers for both yeast and mycelial antigens were negative, but immunodiffusion antibody tests for H and M bands were positive. Cerebrospinal fluid was also positive for *Histoplasma* antigen. Results of magnetic resonance imaging of the patient’s brain were normal for age.

Because the patient’s condition met diagnostic criteria for both HLH and PDH, she was given etoposide, dexamethasone, and liposomal amphotericin B (LAmB), according to treatment guidelines for both conditions (*3*,*4*). Supportive care included transfusion of packed red blood cells, platelets, cryoprecipitate, and fresh frozen plasma. Her fever resolved after 10 days, and other symptoms resolved after 2 weeks. After 16 days of hospitalization, she was discharged to complete a 6-week course of LAmB with a plan to transition to oral itraconazole and complete 1 year of therapy, according to guidelines of the Infectious Diseases Society of America (*4*).

HLH is a rare disease, characterized by impaired function of natural killer and cytotoxic T-lymphocytes that results in an unchecked inflammatory response. If not treated promptly, HLH can progress rapidly to multiorgan failure and death (*3*). HLH is categorized into familial or secondary forms. Familial HLH is caused by mutations in >1 of the genes required for perforin-dependent lymphocyte cytotoxicity; secondary HLH occurs in a person who does not have genetic risk factors (*3*). Both forms can be triggered by infection or malignancy (*3*).

Diagnosis of HLH is based on clinical assessment (Table); no definitive diagnostic test exists (*6*). Initial treatment of HLH involves the use of corticosteroids, etoposide, or other drugs to block the hyperinflammatory response and specific therapy for the inciting infection if available (*7*). In some cases, in which a treatable inciting infection is identified, antimicrobial drug therapy alone might be sufficient. However, concurrent immunosuppressive therapy is usually recommended, especially for patients who are critically ill or whose condition is clinically deteriorating (*3*).

**Table:** Diagnostic criteria for HLH*

Criterion	HLH reference	Patient in this report
Fever	≥38.5°C	40°C†
Splenomegaly	Present	Present†
Cytopenia, 2 of 3 lineages		
ANC, cells/mm^3^	<1,000	1,500
Hemoglobin, g/dL	<9	5.9†
Platelet count, × 10^3^/μL	<100	11†
Low fibrinogen, mg/dL	<150	78†
or		
High triglycerides, mg/dL	>265 fasting	378†
Hemophagocytosis	Present	Present†
NK cell activity	Low or absent	Normal
Elevated ferritin, ng/mL	>500	317 (max 1,218)†
Soluble IL-2 receptor, pg/mL	>2,400	21,530†

*ANC, absolute neutrophil count; HLH, hemophagocytic lymphohistiocytosis; IL-2, interleukin-2; NK, natural killer.  †Criteria met by this patient. The HLH-2004 diagnostic criteria require either a molecular/genetic diagnosis consistent with HLH or fulfillment of 5 of the 8 criteria shown. Adapted from (*5*).

Many clinical manifestations of disseminated histoplasmosis, including prolonged fever, hepatosplenomegaly, pancytopenia, and coagulopathy, overlap with those of HLH. Because of the similarity in manifestations, differentiating these 2 conditions is challenging without specialized testing. Furthermore, laboratory tests specific for HLH, such as measuring soluble interleukin-2 receptor, have not been investigated in patients with isolated PDH, so whether they distinguish between the 2 conditions is unclear. To add further complexity, coexisting histoplasmosis and HLH has been described in several adult patients (*5**,**8*–*10*). However, whether HLH identification and adjunctive immunosuppressive therapy leads to improved outcomes in this situation is unknown.

On the basis of our investigation of this infant with HLH and PDH, we recommend that all infants exhibiting HLH in *Histoplasma*-endemic regions be assessed for histoplasmosis. In addition, the similarity with the clinical features of PDH and the difficulty in diagnosis of HLH without specialized testing raise the question of whether a large number of infants with PDH would also meet the diagnostic criteria for HLH. If so, currently poor outcomes of PDH might be related to co-existing HLH with failure to control the inflammatory response, and the outcomes could be improved by diagnosis and simultaneous treatment of both conditions. Further research is needed to investigate this phenomenon.

Technical Appendix Wright-Giemsa stain of the bone marrow biopsy specimen of a 6-month-old girl with hemophagocytic lymphohistiocytosis and progressive disseminated histoplasmosis.
