# Supplementary material for: Hemophagocytic Lymphohistiocytosis and Progressive Disseminated Histoplasmosis
Source: Emerg Infect Dis. 2016 Jun;22(6):1119–21. doi: 10.3201/eid2206.151682 (PMC4880106; doi:10.3201/eid2206.151682)
Supplement: Technical Appendix — Wright-Giemsa stain of the bone marrow biopsy specimen of a 6-month-old girl with hemophagocytic lymphohistiocytosis and progressive disseminated histoplasmosis. [file 15-1682-Techapp-s1.pdf]

Article DOI: http://dx.doi.org/10.3201/eid2206.151682

Hemophagocytic Lymphohistiocytosis and Progressive Disseminated Histoplasmosis

Technical Appendix

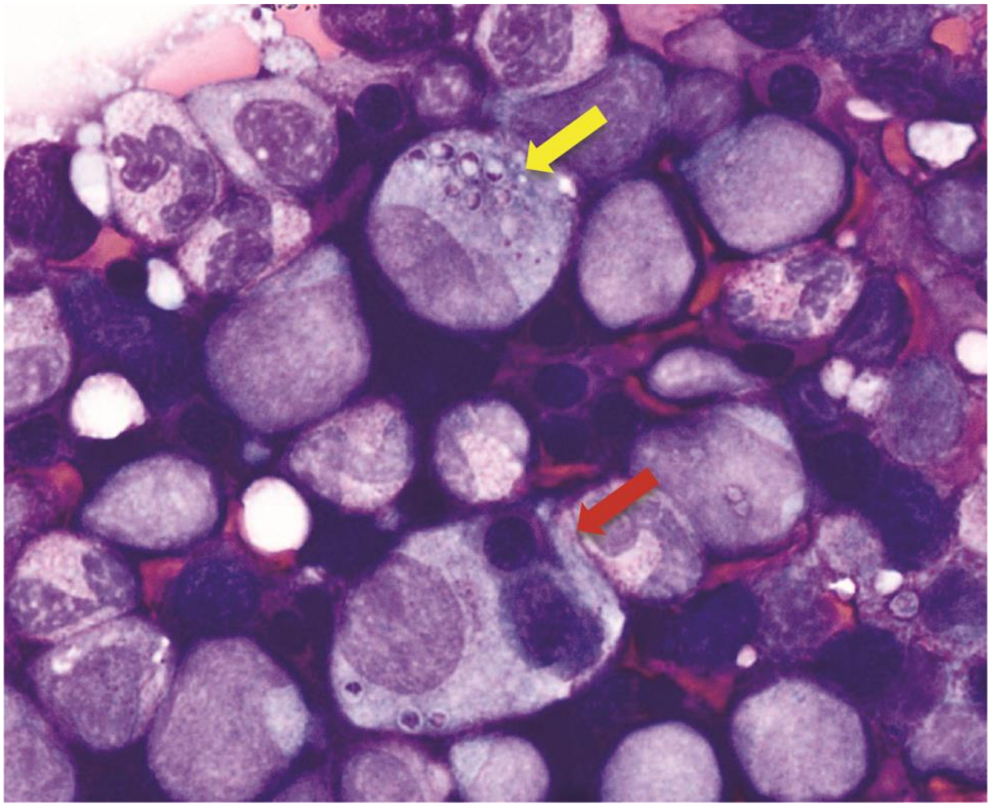

Technical Appendix Figure. Wright-Giemsa stain of the bone marrow biopsy specimen of a 6-month-old girl with hemophagocytic lymphohistiocytosis and progressive disseminated histoplasmosis. Imprints show cytoplasm-rich (activated) macrophages with hemophagocytosis (black arrow) and intracellular organisms (white arrow). Original magnification ×100. A color version of this figure is available online (http://wwwnc.cdc.gov/EID/article/22/6/15-1682-F1.htm).
